# Gaucheroma of Mesenteric Lymph Nodes and Their Ultrasound Appearance: A Case Report

**DOI:** 10.5334/jbsr.3184

**Published:** 2023-05-30

**Authors:** Thomas Saliba, Paolo Simoni, Aurélie Empain, Alessandro De Leucio

**Affiliations:** 1 ULB, BE; 2 Hopital Universitaire Des Enfants Reine Fabiola, BE

**Keywords:** Gaucher’s disease, Gaucheroma, lymph node, ultrasound, diagnosis

## Abstract

Gaucher disease represents the largest lysosomal storage disease group worldwide. Possible complications include the development of Gaucheromas, pseudotumors resulting from an accumulation of Gaucher cells. Gaucheromas can affect the liver, spleen, bones, and lymph nodes. Descriptions of the appearance of lymph node gaucheromas exist for computed tomography (CT) and magnetic resonance imaging (MRI) but not, to our knowledge, of their ultrasound characteristics. We present the case of a four-year-old boy with Gaucher disease with lymph node Gaucheromas, discovered during a routine follow-up, and present their ultrasound characteristics. We describe characteristic ultrasound findings of non-B-cell lymphomas and Gaucheroma lymph nodes in comparison.

**Teaching point:** Lymph node Gaucheromas have a characteristic ultrasound appearance and should be searched for in Gaucher’s patients.

## Introduction

Gaucher disease (GD) patients represent the largest lysosomal storage disease group worldwide, the disease being caused by a recessive GBA gene mutation [1]. Any new focal lesion in a GD patient should be carefully examined due to their increased risk of developing complications such as multiple myeloma or lymphoma [1]. If a mass is detected, however, Gaucheromas must also be considered. Gaucheromas are a rare manifestation of GD caused by the accumulation of Gaucher cells resulting in a pseudotumor [1]. Gaucheromas have been found to involve the liver, spleen, bones, and lymph nodes [1,2,3,4]. When lymph node involvement occurs, the preferential location is mesenteric lymph nodes, though liver, mediastinal, cervical, and axillary Gaucheromas have also been reported [1,5]. Gaucheromas remain rare, with less than 30 reported cases [1,5]. Nevertheless, Gaucheromas can have clinically important repercussions due to their ability to cause protein-losing enteropathy [5].

## Case History

**Figure 1:**
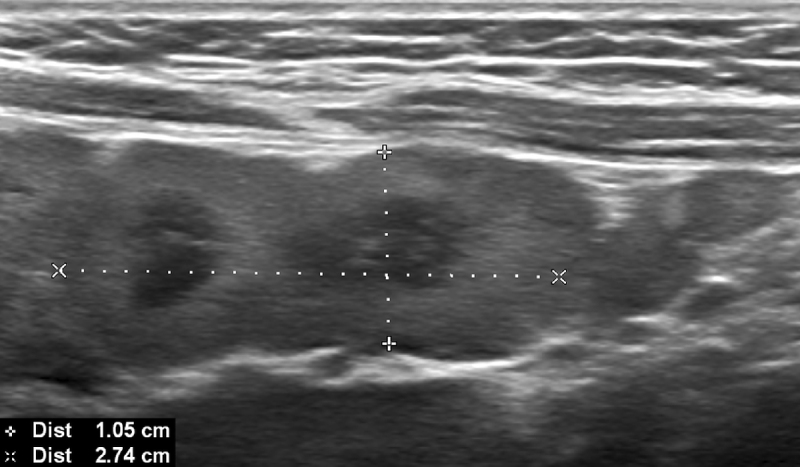
Greyscale ultrasound image demonstrating confluent lymph nodes with a supra-centimetric small axis.

**Figure 2:**
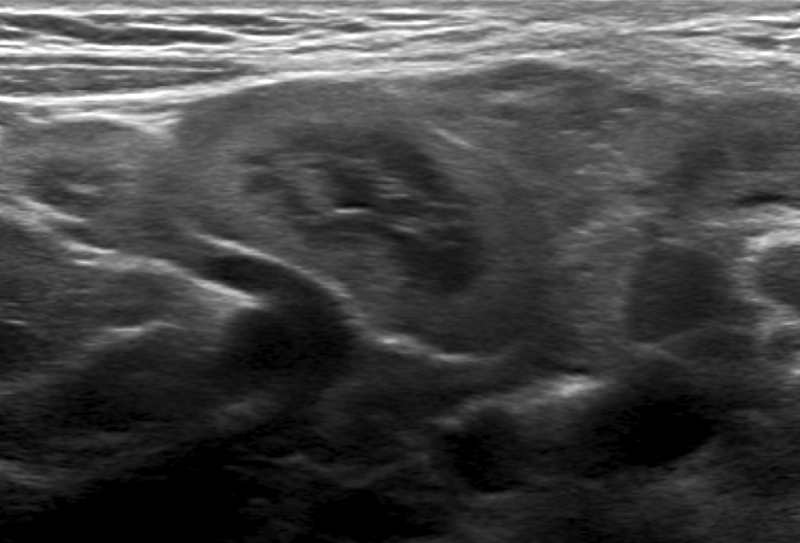
Greyscale ultrasound image demonstrating the distinctive hyperechogenic cortex and hypoechogenic medulla.

**Figure 3:**
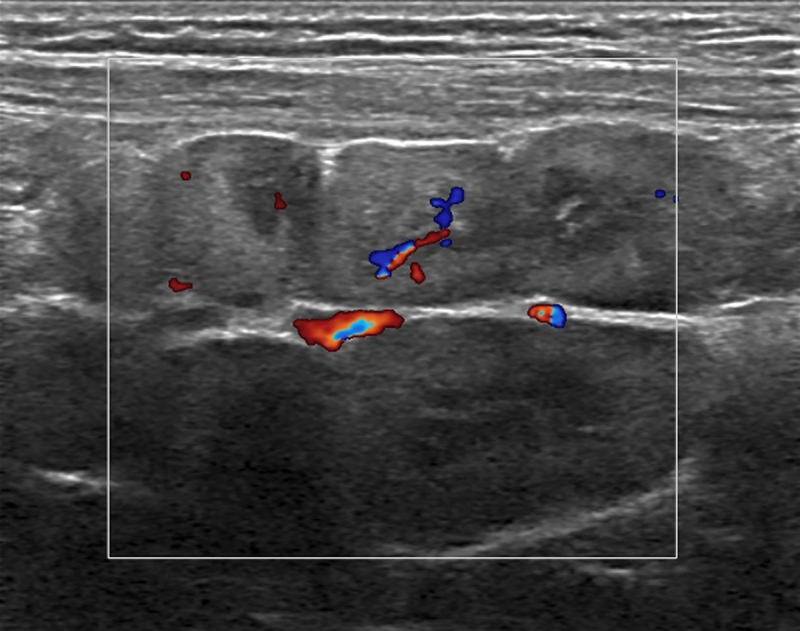
Colour Doppler ultrasound image demonstrating hilar vascularisation of the lymph nodes.

A four-year-old boy with a known GD presented for his annual ultrasound. Upon examination large mesenteric lymph nodes were discovered, with an unusual confluent appearance, hyperechogenic cortex, and hypoechogenic medulla (Figures 1 and 2). The lymph nodes also presented with normal hilar vascularisation (Figure 3). Further testing was done to rule out neoplastic or infectious causes for the lymphadenopathy, resulting in the conclusion that they were Gaucheromas. The appearance of lymphadenopathy in a patient with GD is a known complication, and therefore finding lymph node Gaucheromas is not unexpected. An attempt to characterise the vascular resistance was made but was impossible due to the child’s agitation. Magnetic resonance imaging (MRI) confirmed the findings. At the time of writing the patient’s Gaucheromas were stable on his follow-up exam (seven months after their first appearance), despite imiglucerase treatment, though this is to be expected without an increase in dose.

## Comments

Gaucheromas are a rare complication of GD, with several known complications such as protein-losing enteropathy, alongside further complications it engenders, as well as obstructions and compressions due to the mass effect of the lymphadenopathies [5]. Currently, most Gaucheromas are detected in paediatric patients, with 75% being discovered in patients under 10 years old [5].

The broad lines of the radiological description of Gaucheromas have been established in the literature regarding their appearance in computed tomography scans and MRI, with the lesions being described by Ye et al. as large lymphadenopathy with calcifications visible in CT and no specific MRI description to our knowledge [3]. Additionally, no papers have undertaken a thorough description of the ultrasound characteristics, despite it being the exam of choice, being non-irradiating and requiring no sedation and having reproducibility issues as its only disadvantage. In our patient, the lymph node Gaucheromas, when studied using ultrasound, appear as large confluent masses with a hyperechogenic cortex and a hypoechogenic medulla, reminiscent of the kidney of a neonate. This, to our knowledge, is the first description of the ultrasound appearance of lymph node Gaucheromas. Due to the propensity of Gaucher patients to develop B-Cell non-Hodgkin’s as well as Hodgkin’s lymphomas, it is important to be able to differentiate the two entities (see Table 1) [6,7].

**Table 1:** Juxtaposition of lymph node appearances in Gaucheromas and lymphomas [6].


LYMPH NODES CHARACTERISTICS	GAUCHEROMA	LYMPHOMA

**Size**	>1cm	>1cm

**Shape**	Irregular	Regular, round

**Echogenicity**	Hyperechogenic cortex, hypoechogenic medulla	Hypoechogenic, sometimes lacking hilus

**Vascular distribution**	Predominantly hilar	Hilar and peripheral


## Conclusion

Lymph node Gaucheromas are a rare but not unknown complication in patients with GD. We presented the case of a four-year-old in whom large mesenteric lymphadenopathy was detected during a routine abdominal ultrasound. We provide an ultrasound description of lymph node Gaucheromas, with comparison to expected findings in lymphoma, which has an increased incidence in Gaucher sufferers, with lymph node involvement.
